# Morphological variation, management and domestication of ‘maguey alto’ (*Agave inaequidens*) and ‘maguey manso’ (*A. hookeri*) in Michoacán, México

**DOI:** 10.1186/1746-4269-10-66

**Published:** 2014-09-16

**Authors:** Carmen Julia Figueredo, Alejandro Casas, Patricia Colunga-GarcíaMarín, Jafet M Nassar, Antonio González-Rodríguez

**Affiliations:** Centro de Investigaciones en Ecosistemas, Universidad Nacional Autónoma de México, Campus Morelia, Apartado Postal 27-3 (Santa María de Guido), Morelia, Michoacán 58190 Mexico; Centro de Investigación Científica de Yucatán, A.C. Calle 43 No. 130, Colonia Chuburná de Hidalgo, Mérida, Yucatán CP 97200 México; Centro de Ecología, Instituto Venezolano de Investigaciones Científicas, Carretera Panamericana km 11, Edo, Miranda Venezuela

**Keywords:** Domestication, Mescal agave, Phenotypic variation, Plant management, Pulque

## Abstract

**Background:**

*Agave inaequidens* and *A. hookeri* are anciently used species for producing the fermented beverage ‘pulque’, food and fiber in central Mexico. *A. inaequidens* is wild and cultivated and *A. hookeri* only cultivated, *A. inaequidens* being its putative wild relative. We analysed purposes and mechanisms of artificial selection and phenotypic divergences between wild and managed populations of *A. inaequidens* and between them and *A. hookeri*, hypothesizing phenotypic divergence between wild and domesticated populations of *A. inaequidens* in characters associated to domestication, and that *A. hookeri* would be phenotypically similar to cultivated *A. inaequidens*.

**Methods:**

We studied five wild and five cultivated populations of *A. inaequidens*, and three cultivated populations of *A. hookeri*. We interviewed agave managers documenting mechanisms of artificial selection, and measured 25 morphological characters. Morphological similarity and differentiation among plants and populations were analysed through multivariate methods and ANOVAs.

**Results:**

People recognized 2–8 variants of *A. inaequidens*; for cultivation they select young plants collected in wild areas recognized as producing the best quality mescal agaves. Also, they collect seeds of the largest and most vigorous plants, sowing seeds in plant beds and then transplanting the most vigorous plantlets into plantations. Multivariate methods classified separately the wild and cultivated populations of *A. inaequidens* and these from *A. hookeri*, mainly because of characters related with plant and teeth size. The cultivated plants of *A. inaequidens* are significantly bigger with larger teeth than wild plants. *A. hookeri* are also significatly bigger plants with larger leaves but lower teeth density and size than *A. inaequidens*. Some cultivated plants of *A. inaequidens* were classified as *A. hookeri*, and nearly 10% of *A. hookeri* as cultivated *A. inaequidens*. Wild and cultivated populations of *A. inaequidens* differed in 13 characters, whereas *A. hookeri* differed in 23 characters with wild populations and only in 6 characters with cultivated populations of *A. inaequidens*.

**Conclusions:**

Divergence between wild and cultivated populations of *A. inaequidens* reflect artificial selection. *A. hookeri* is similar to the cultivated *A. inaequidens*, which supports the hypothesis that *A. hookeri* could be the extreme of a domestication gradient of a species complex.

## Background

Domestication is an evolutionary process mainly guided by artificial selection [1], and also influenced by other evolutionary forces guided by humans [2]. This process is modelled by human culture, social needs and technology, but it is strongly influenced also by the variable nature of ecosystems and populations of the managed organism. Throughout time domestication may determine morphological, physiological or reproductive divergences among wild and managed populations of organisms [3, 4]. However, although in a population artificial selection may reduce the variation of managed organisms, the result of the general process is the generation of divergence and variation among pools of organisms and it is, therefore, a diversifying process as illustrated by Darwin [1].

It is defined as ‘domestication syndrome’ the morphological and physiological features that are similar among different domesticated species, because of similar principles of artificial selection (evolutionary convergence) favouring particular characteristics interesting to humans [5]. These features have similar tendencies in dozens of domesticated species and can be identified when domesticates are compared with their wild relatives and with other domesticates [6–8]. Mesoamerica is an important centre of domestication, and wild relatives of numerous domesticated plant species occur in its territory [6]. Therefore, the area allows favourable conditions for analysing comparatively evolutionary trends of wild and domesticated taxa, which is in turn valuable information for studying how domestication occurred in the past and how it is currently occurring. A high variety of wild and domesticated varieties of worldwide important plant genetic resources such as maize, beans, squashes and pumpkins, tomatoes, cotton, cocoa, chili peppers, occur in this area [6]. In addition, there is a high variation of other species secondarily important like numerous fruit trees, prickly pears, columnar cacti, and agaves [9–12]. Finally, in this region occurs a great diversity of plant species utilized by Mexican peoples and that currently is in a continuous gradient of phases of domestication. The traditional forms of management are ongoing processes with a great diversity of environmental and cultural conditions. Their mechanisms and evolutionary trends are highly diverse, much higher than those described for the most studied crops; therefore, their study offers the possibility to enrich principles and theory of domestication mechanisms and trends [9–14].

Agaves are plants endemic to the Americas, and the species that constitute this plant group are mainly distributed in arid and semiarid zones, tropical dry and temperate forests [15]. The genus *Agave* comprises nearly 200 species that are generally key components in the ecosystems in which they form part [15]. Dozens of agave species have been of high cultural and economic importance for humans that have interacted with them, particularly for the indigenous cultures of Mesoamerica, which have utilized agaves for more than 10000 years [16–18]. Several species have been domesticated since they are important sources of food, fibers, medicines and beverages [19], as it has been documented by both archaeological and ethnobotanical studies [17–19]. It is known that currently nearly 102 taxa (species and intraspecific taxa) of agaves are used in México with different purposes, mainly as food, and for obtaining fermented and distilled alcoholic beverages, construction material, and fodder [19].

Only nine of the agave species recognized as domesticates have been studied analysing the consequences of the process of domestication on phenotypic divergence. Among the species considered as the putative ancestor of *A. fourcroydes* Lem., the species known as henequén widely used in Yucatán for extracting fibre [20, 21]. Also *A. angustifolia*, *A. rhodacantha* Trel. and *Agave tequilana* Weber in the central-western region of Mexico, used for producing mescal and tequila [22–24]. The morphological variation of *A. salmiana* Otto ex. Salm, *A. macroculemis* Tod. and *A. mapisaga* Trel. has been studied in the central-northern area of Mexico [25], and more recently *A. parryi* Engelm. in south-eastern Arizona [26].

The main trends of phenotypic divergence between wild and cultivated populations of agaves identified as associated to artificial selection are: i) Larger size or higher biomass of the whole plant or of the particularly useful parts (leaves, fibre), ii) higher concentration of sugar in the plant tissue and sap, iii) higher production of sap, iv) less spikiness (smaller spines or less density of these structures), and v) other aspects that make easier the propagation of agave, for instance lower production of caustic secondary compounds that irritate human skin [20–25]. Although sexual reproductive parts (flowers, inflorescences, capsules and seeds) have been analysed in the studies available, these have been found to be irrelevant for domestication analyses, because the main targets of artificial selection in agaves are the vegetative parts [21].

In central-western Mexico, two closely related agave species: *A. inaequidens* Koch and *A. hookeri* Jacobi are widely used, probably since thousands of years ago for extracting the sap ‘agua miel’ to produce ‘pulque’ and other plant parts consumed as food. *A. inaequidens* is also used for extracting fibre [27], and according to historical sources, this agave is used for producing mescal from approximately 400 years ago. Our research group has observed in the field different types of management of wild, silvicultural and cultivated populations, which constitute a gradient of management intensity. Artificial selection can be currently occurring, and it is possible to suppose that it occurred in the past since people favour the reproduction of particular phenotypes through different management practices. These are the cases of phenotypes considered to have good quality as food, for pulque and mescal production, and fibre extraction. In this study, we explored whether artificial selection is operating, and what are the purposes and mechanisms of this selection in *A. inaequidens* and *A. hookeri*. In addition, we analysed if artificial selection has determined divergence between wild and cultivated populations, and how much divergence the process has determined. We particularly expected that as more intense the artificial selection, the higher phenotypic divergence between wild and domesticated populations would be.

Hitherto, *A. hookeri* has been recorded only under cultivation forming live fences but not in the wild. According to [28], very few records of this species have been published, and all of them are from the state of Michoacán. Morphologically, this species is similar to *A. inaequidens*, and taxonomists have proposed that these taxa are closely related [28]. These species occur simpatrically and in general, it is possible to distinguish them phenotypically, particularly because of differences in size and color of leaves, size of the main terminal spine and flowers size, but according to [28] it is also possible to suspect hybridization among these taxa since it is possible to find individual plants with intermediate characters. This fact, leads to a third main question of our study: How divergent are among themselves *A. inaequidens* and *A. hookeri*? It has been proposed [28] that *A. inaequidens* could be a putative wild ancestor of *A. hookeri*, which under this hypothesis would be in an extreme of morphological variation associated to artificial selection. Our study, therefore, aspires to provide information to contribute to test such a hypothesis.

## Materials and methods

### Species studied

*Agave inaequidens* (Agavoideae, Asparagaceae *sensu* [29]) is endemic to Mexico, naturally growing along the Trans-Mexican Volcanic Belt in pine and pine-oak forests [28]. It is a monocarpic, medium to large sized, short-stemmed, and openly spreading plant species. The leaves are variable sized, broadly or narrowly lanceolate to oblanceolate, light green to yellow-green, the margin undulate to repand and crenate [28]. The distinguishing characteristic is the dimorphism of teeth (successively shorter and larger) which are castaneous to dark brown. The main spine is stout broadly, deeply channeled above and dark brown. The panicles are 5–8 m tall. The flowers are yellow, protandrous, xenogamous, and visited by bats and other diurnal animals [30]. The capsules are oblong, stipitate, rounded to apiculate at the apex, brown; the seeds are hemispherical, shiny black, dispersed by wind [28, 30].

**Figure 1:**
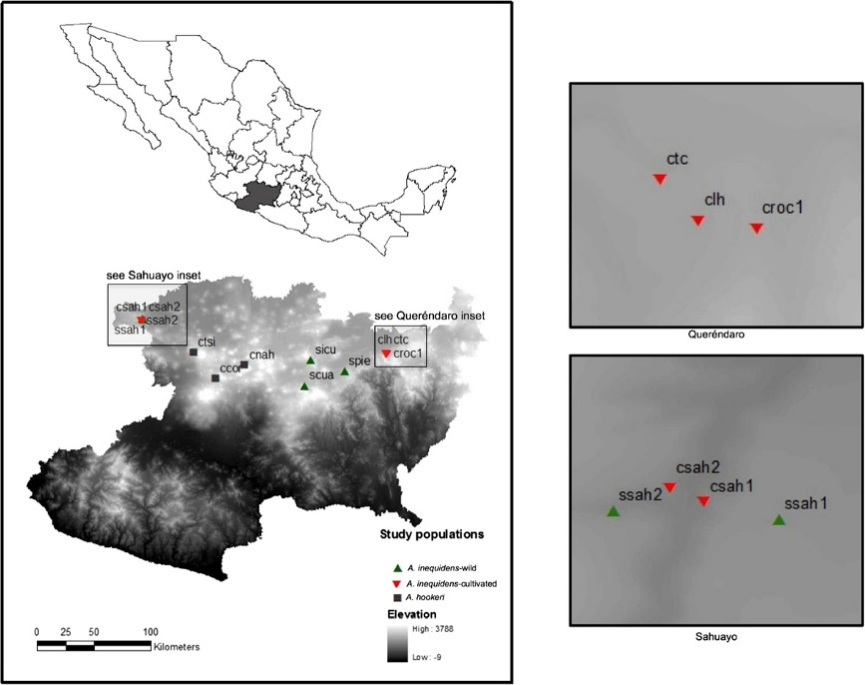
**Localization of the populations of** ***Agave inaequidens*** **(green triangle symbol are wild, red down pointing triangle symbol are cultivated) and A.** ***hookeri*** **(grey square symbol)** ***s*** **tudied in Central Occidental Mexico.**

**Figure 2:**
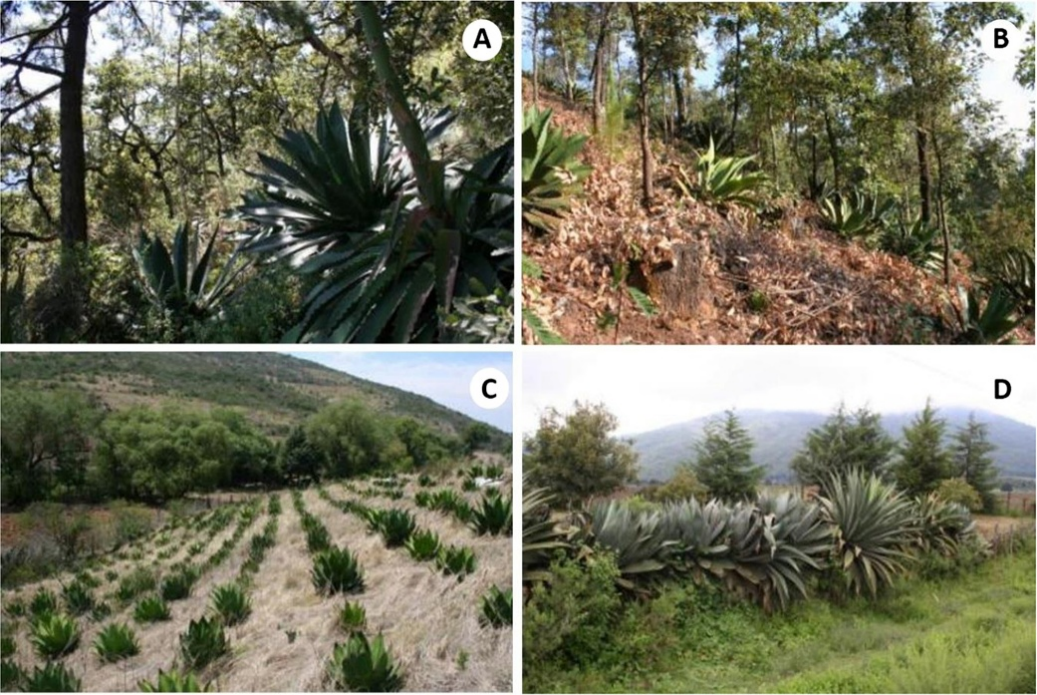
**Aspects of the populations of** ***Agave inaequidens*** **and** ***A. hookeri*** **studied. A)** Wild population of *A. inaequidens* in oak forest **B)** *In situ* managed population of *A. inaequidens* in disturbed pine-oak forest, **C)** Cultivated population of *A. inaequidens* and **D)** Population of *A. hookeri* in live fence (photos by Ignacio Torres).

This species has several uses; historically it was consumed as food and its sap extracted for drinking fresh (‘agua miel’) or fermented (‘pulque’) [28]. More recently, it is used for producing the distilled spirit mescal [31]. *A. inaequidens* has the common name of ‘maguey alto’ or ‘maguey bruto’, terms that refer to its size and the caustic condition of its tissue, respectively, because of the presence of saponins and other secondary metabolites causing dermatitis [28]. Moreover, its fiber has been used in the region of San Miguel Cuyutlán, Jalisco, for the manufacture of ropes or cords used in horsemanship [27]. In the state of Michoacán (Figure 1), it is possible to see this species in a gradient of management intensity with populations in wild habitats as part of natural forests, but also under silvicultural or ‘*in situ*’ management, through which people let some individuals standing when the forest is cleared, and deliberately propagate agaves in the cleared areas in order to increase their population density. Some people also practice cultivation of this agave species out of its natural habitat (*ex situ* cultivation) (Figure 2).

*Agave hookeri* belongs to the Crenatae group, and it is recognizable by its large size, glaucous leaves with a strong tongue-like projection from the spine base, short tube and very long tepals [28]. This species is cultivated in rows forming live fences, which have the double function of protecting a plot and producing pulque. *A. hookeri* is closely related to *A. inaequidens* and they may be hard to be distinguished. Both species show variation in leaf color, shape and size of leaf and armature [28]. Gentry [28] suggested that mixing between species involves not only mixed plantings, but also hybridization. The phylogenetic relationships between these species are still unclear, but according to Gentry [28], the most likely wild ancestor of *A. hookeri* is *A. inaequidens.*

### Study area

The study was conducted in 13 populations located in the state of Michoacán, Mexico: five wild and five cultivated populations of *A. inaequidens*, and three populations of *A. hookeri*. Three were wild populations located in the municipalities of Pátzcuaro, Quiroga, and Morelia (Figure 1) growing in pine-oak, pine, and oak forests at elevations of 2400 to 2600 m, dominated by *Quercus castanea* Née*, Q. candicans* Née*, Q. laeta* Liebm*, Q. crassipes* Humb. & Bonpl*, Q. rugosa* Née, *Pinus ponderosa* Douglas ex. Lawson, *P. edulis* Engelm, and *Arbutus unedo* L. Two wild populations were located in the municipality of Sahuayo, one of them growing in a subtropical scrub at 1900 m elevation dominated by *Euphorbia tanquehuete* V.W. Steinm. & Dorsey, *Bursera fagaroides* (Kunth) Engl., *Eysenhardtia polystachya* (Ortega) Sarg. and *Ipomea murucoides* Roem. & Schult*.*, and the other population is located in a pastureland dominated by *Mimosa* sp. For comparing morphological trends of variation, we studied three populations cultivated in orchards at Queréndaro, where agaves were recorded growing together with several species of fruit trees and other agave species (*A. tequilana* and *A. cupreata* Trel. & Berger). Two additional populations were studied in orchards of the municipality of Sahuayo. Three cultivated populations of *A. hookeri* were studied in Uruapan, Tingüindín and Nahuatzen, which were live fences with few plants of this latter species.

### Ethobotanical assessment

We visited each municipality and population, conducting open interviews to the managers and owners of the lands, and direct observations of wild populations of *Agave inaequidens* and orchards where the two agave species are cultivated. Additional semi-structured interviews were conducted to document targets and mechanisms of artificial selection, as well as the areas the cultivated plants come from.

### Morphological variation of the plant

A total 25 morphological characters (Table 1) were measured in samples of plants of each population; ten of these are ratios that reflect the relationship of structures under artificial selection according to [21]. We measured two to three leaves of each individual plant; the number of teeth was measured on one side of each leaf. Through multivariate statistical methods, we analyzed patterns of morphological similarity and differentiation among individual plants within populations and among populations. We used Cluster Analysis (CA) and Principal Components Analysis (PCA) to classify the populations sampled according to the average values of the morphological features studied. Similarly, we used PCA and Discriminant Function Analysis (DFA) [32] to classify the individual plants of all the populations sampled, in order to explore whether or not the morphological similarities relate to their management type and the species studied.

**Table 1:** **Characters evaluated in the variation analysis, mean values ± s. e in wild and cultivated population of** ***Agave inaequiens*** **and population of** ***A. hookeri***

Character	***Agave inaequidens***		***Agave hookeri***	PC1	PC2	DF1	DF2
	Wild populations	Cultivated populations					
Plant total lenght (PTL)**	126.348 ± 3.624 **A**	148.327 ± 2.962 **B**	196.417 ± 5.003 **C**	**0.969**	0.157	**0.503**	0.229
Stem lenght (SL)*	44.941 ± 1.183 **A**	47.008 ± 1.407 **A**	53.1500 ± 2.0638 **B**	0.460	0.219	0.099*	-0.004
Diameter of the plant 1 (D1)**	221.373 ± 5.836 **A**	199.990 ± 5.560 **B**	291.333 ± 7.297 **C**	**0.834**	-0.273	0.124	**0.446**
Diameter of the plant 2 (D2)**	220.246 ± 5.682 **A**	203.092 ± 5.372 **B**	279.333 ± 6.518 **C**	**0.825**	-0.286	0.084	0.355
Leaf lenght (LL)**	97.997 ± 2.540 **A**	106.385 ± 2.220 **A**	156.784 ± 4.132 **B**	**0.973**	0.058	**0.452**	**0.437**
Leaf width at middle (LW)**	16.522 ± 0.422 **A**	20.861 ± 0.383 **B**	20.783 ± 0.450 **B**	0.595	0.471	**0.434**	-0.136
LL/LW**	6.076 ± 0.130 **A**	5.187 ± 0.112 **B**	7.846 ± 0.349 **C**	0.538	-0.332	0.043	**0.526**
LL/SL**	2.246 ± 0.055 **A**	2.453 ± 0.087 **A**	3.121 ± 0.099 **B**	**0.908**	0.034	**0.325**	0.312
Terminal thorn length (TTL)**	3.405 ± 0.060 **A**	4.039 ± 0.086 **B**	5.038 ± 0.157 **C**	0.446	0.466	**0.457**	0.229
Terminal thorn width at the base (TTW)**	0.588 ± 0.013 **A**	0.732 ± 0.017 **B**	0.761 ± 0.028 **B**	-0.356	-0.213	**0.363**	-0.099
TTL/TTW**	5.993 ± 0.154 **A**	5.756 ± 0.160 **A**	7.132 ± 0.345 **B**	0.003	0.108	0.063	0.245
TTL/LL*	0.038 ± 0.001 **A**	0.039 ± 0.001 **A**	0.033 ± 0.001 **B**	**-0.779**	0.216	-0.091	-0.142
Total number of teeth (TEE)**	60.154 ± 2.059 **A**	61.153 ± 2.008 **A**	74.108 ± 1.682 **B**	**0.828**	-0.269	0.165	0.177*
TEE/LL**	0.621 ± 0.014 **A**	0.584 ± 0.018 **A**	0.483 ± 0.012 **B**	-0.591	-0.477	-0.187	-0.125
Number of teeth in 10 cm^2^ (TEE10)**	6.244 ± 0.190 **A**	5.138 ± 0.193 **B**	4.517 ± 0.180 **B**	**-0.691**	-0.611	-0.276	0.044
Teeth length 1 (LTEE1)**	0.572 ± 0.016 **A**	0.752 ± 0.020 **A**	0.660 ± 0.028 **B**	-0.013	**0.956**	**0.305**	-0.232
Teeth length 2 (LTEE2)*	0.306 ± 0.014 **A**	0.388 ± 0.020 **B**	0.385 ± 0.023 **B**	0.128	**0.892**	0.206	-0.063
LTEE1/LL**	0.006 ± 0.0003 **A**	0.007 ± 0.0003 **B**	0.004 ± 0.0002 **C**	**-0.863**	0.495	-0.05	-0.348
LTEE2/LL*	0.003 ± 0.0001 **A**	0.004 ± 0.0002 **A**	0.003 ± 0.0002 **B**	**-0.778**	0.477	-0.028	-0.202
Teeth width 1 (WTEE1)**	0.842 ± 0.021 **A**	1.065 ± 0.031 **B**	1.086 ± 0.038 **B**	0.504	**0.754**	**0.384**	-0.082
Teeth width 2 (WTEE2)*	0.454 ± 0.021 **A**	0.569 ± 0.036 **B**	0.556 ± 0.038 **B**	0.076	**0.779**	0.188	-0.039
LTEE1/WTEE1*	0.694 ± 0.015 **A**	0.725 ± 0.016 **A**	0.629 ± 0.037 **B**	**-0.757**	0.139	-0.039	-0.089
LTEE2/WTEE2	0.732 ± 0.024 **A**	0.756 ± 0.031 **A**	0.678 ± 0.033 **A**	**-0.876**	0.288	-0.015	-0.092
Distance between teeth (DTEE)*	0.964 ± 0.048 **A**	1.214 ± 0.081 **B**	1.343 ± 0.080 **B**	0.420	0.523	0.164	-0.055
DTEE/LL*	0.011 ± 0.0005 **AB**	0.0117 ± 0.0008 **B**	0.0088 ± 0.0006 **A**	-0.390	0.463	-0.033	-0.182

All measurements in cm except Total number of teeth (TEE) and Number of teeth in 10 cm^2^ (TEE10) these amount. Different capital letters in bold among populations indicate significant differences according to ANOVA and Tukey test (*p ≤ 0.05, (**p ≤ 0.01). The last columns show eigenvectors of the first (PC1) and second (PC2) principal components according to PCA, and the Discriminant Functions 1 and 2.

Due to differences associated to character type and measurement units, we standardized the data matrix using the algorithm *Y*_*0*_ *= (Y-a)/b*; where *Y*_*0*_ is the standardized value, *Y* is the real value of a character state, *a* is its average and *b* its standard deviation [32]. PCAs and CA were performed with NTSys 2.02 [33], and DFA through IBM-SPSS Statistics 22. CA based on a similarity calculated a cophenetic correlation matrix and the *r*-value. The PCAs were performed based on similarity matrixes using the coefficient of variance-covariance. Eigenvectors allowed identifying morphological characters with higher meaning to classify populations and significance of differences among wild and cultivated populations of *A. inaequidens* and populations of *A. hookeri*. We performed one-way ANOVAs through IBM-SPSS Statistics 22 in order to identify how morphological characters studied differed between the populations studied.

## Results

### Ethnobotanical studies

The main uses recorded for *A. inaequidens* were the consumption of the escape tissue as food, as well as the extraction of the sap (‘agua miel’) for producing ‘pulque’, and the extraction of stems for preparing mescal. It is popularly called ‘maguey alto’ (‘tall agave’), ‘maguey bruto’ (‘brutish agave’), and ‘mescal’. For producing mescal, people choose individuals of wild populations which have been previously ‘capados’, that is, their escapes were cut before emerging (a state of development locally called ‘jorras’), in order to avoid the production of flowers. ‘Jorras’ are recognized as the development stage in which agaves produce more sap with higher sugar concentration, which is favourable for fermentation and pulque production; also, concentration of sugar is higher in the stem tissue, which favours the quality and quantity of mescal. The people interviewed recognized two to eight varieties of *A. inaequidens* in the wild populations (ten varieties in total), all of them based on differences in plant size, colour, and form and leaf size (Table 2). For producing mescal people do not take into account the varieties, they collect all the individuals available in the stage of ‘jorras’. However, all people interviewed had rustic nurseries and cultivated plants of this agave at their homes, and for cultivation they take into account the characteristics of the plants providing propagules, as explained below. However, the extraction of agaves from forests is a common practice, particularly when plantations are not still ready for harvesting and because plantations are insufficient for their purposes of mescal production. Therefore, the main purpose of cultivation is to increase the availability of raw matter for mescal production. Plants that are cultivated generally are young plants collected in at least two wild areas where people use to collect plants for producing mescal; these are areas recognized because of the good quality of agaves for producing mescal. In addition, people collect for cultivation seeds of the largest and most vigorous plants from the wild or from other cultivated plants. They sow the seeds in plant beds and the plantlets are then transplanted to orchards, live fences or plantations, artificially selecting the most vigorous plantlets produced. People interviewed affirmed to have not seen shoots or vegetative sprouts produced by this agave in natural populations; but they said that shoots are commonly produced by agaves when plants are manipulated for extracting sap or ‘agua miel’; but, according to their point of view these vegetative shoots have low success for establishment or cultivation.

**Table 2:** **Varieties of** ***A. inaequidens*** **recognized by people of Michoacán, Mexico**

Variety name	Main features	Habitat
‘Maguey chico’	Produce good mescal, but high dermatitis because it has higher concentration of saponins. Its cooked tissue is sweeter than other varieties.	Grow in cleared sunny areas of pine-oak forest (Queréndaro)
‘Maguey grande’	The cooked stem has higher amount of water than other varieties, cause low dermatitis (lower concentration of saponins), less sweet than other varieties	Grow in shaded areas of the pine-oak forests (Queréndaro)
‘Maguey verde’	Small size with light Green leaves	Pine-oak forest (Queréndaro and Quiroga),
‘Maguey cenizo o negro’	Large size with dark green leaves.	Pine-oak forest (Queréndaro and Quiroga),
‘Maguey hoja ancha’	Leaves notoriously wider than other varieties	Pine-oak forest (Queréndaro)
‘Maguey hoja angosta’	Leaves notoriously narrower than other varieties	Pine-oak forest (Queréndaro)
‘Maguey de hojas largas y espina chica’	Leaves long but with smaller terminal spines	Pine-oak forest (Queréndaro)
‘Maguey de hojas cortas y espina grande’	Leaves short but with longer terminal pines	Pine-oak forest (Queréndaro)
‘Maguey bruto mezcal’	Plants with fewer narrower and plain leaves. Produce good mescal	Tropical dry forest (Sahuayo)
‘Maguey bruto chapín’	Plants with abundant, wider chaneled leaves. Used for producing good mescal, and in the process of distillation used as condenser and collector of the distilled mescal	Tropical dry forest (Sahuayo)

The main use of *Agave hookeri* is extraction of ‘agua miel’ (*teri* in P’hurépecha language) for producing ‘pulque’ (*urape* in P’hurépecha). This plant species is called *akamba* (meaning agave in Purhépecha). In the population of the village of Tsirio we found individual plants of *A. hookeri* cultivated as live fences together with plants of *A. inaequidens*, similarly as reported by [28]. After an individual plant of *A. hookeri* has been used for extracting ‘agua miel’, it produces sprouts in the cormo, and these sprouts are utilized for cultivating them in live fences or plots. Few individuals of this species get the sexual reproduction age. According to people, these plants produce capsules and seeds but their seeds, seedlings or plantlets are not collected for sowing or transplanting because are weaker and unsuccessful for establishment.

### Patterns of morphological variation

**Figure 3:**
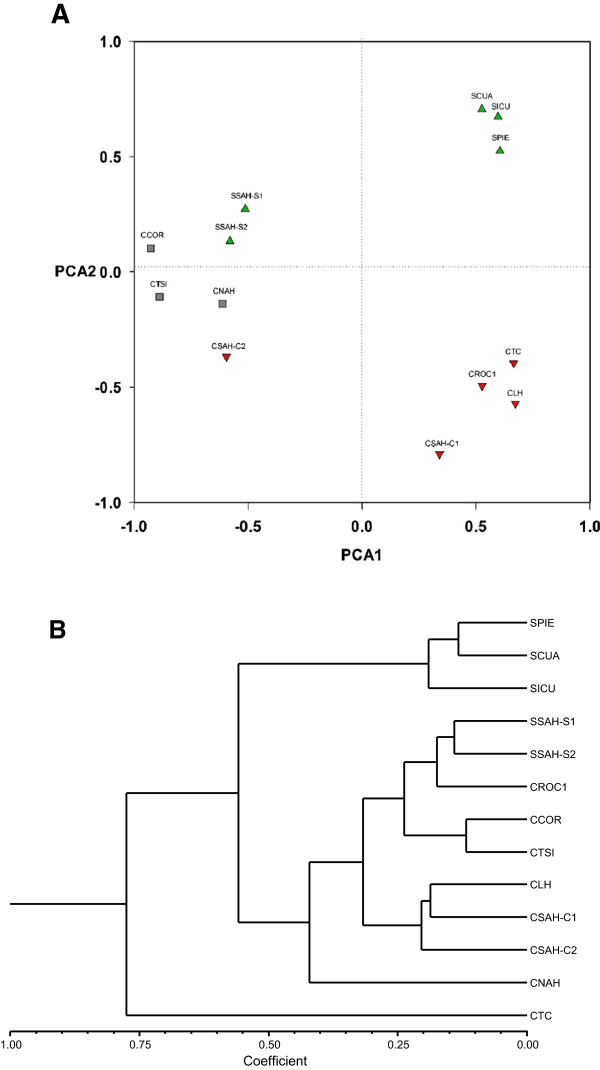
**Classifcation of populations of** ***A. inaequidens*** **and** ***A. hookeri*** **according to the multivariate morphological variation analysed. (A)** Projection of wild (▲ wild) and cultivated (▼ cultivated) populations of *Agave inaequidens* and the populations of *A. hookeri* (■) in the space of the first and second principal components. **(B)** Classification of population wild and cultivated *Agave inaequidens* and population of *A. hookeri* using Cluster Analysis (CA), *r* value 0.8451.

In the PCA classifying populations, the first two principal components explained nearly 62% of the variation, clearly separating the wild populations of *A. inaequidens* at the top section of the plot and the cultivated ones in the lower area of the plot (Figure 3A). This analysis separated clearly the populations of *A. hookeri* at the left of the plot. The eigenvectors indicate that the characters with higher contribution to the first principal component are plant total length (PTL), diameter of the plant 1 and 2 (D1, D2), leaf length (LL), ratio of leaf length and stem length (LL/SL), total number of teeth (TEE), ratio of teeth length and leaf length (LTEE1/LL) and teeth width (WTEE2). The cultivated plants of *A. inaequidens* had generally higher values of these variables, indicating that are larger with larger teeth than the wild individual plants of this species. However, the wild plants had larger diameters, indicating that these plants are shorter than the cultivated ones but their rosettes are more open or extended. Flowers and other reproductive parts analysed were generally similar among both wild and cultivated populations. Individual plants of *A. hookeri* exhibited higher values of plant total length, diameters of the plant, leaf length, ratio of leaf length and stem length, total number of teeth compared with either wild or cultivated plants of *A. inaequidens*, but lower teeth density and size than *A. inaequidens*, which indicates that *A. hookeri* is less armed than *A. inaequidens*. In the second principal component the most relevant characters were teeth length 1 y 2 (LTEE1, LTEE2), ratio of teeth length and teeth width 1 and 2 (LTEE1/WTEE1 and LTEE2/WTEE2). Teeth of the cultivated plants of *A. inaequidens* exhibited higher values than the wild ones, whereas plants of *A. hookeri* had the lowest values (Table 1).

The groups identified by the CA were consistent with those found through PCA (Figure 3B). The cophenetic test showed *r* = 0.89. One branch classified together the cultivated population CTC, and a group of wild populations PIE, ICU and CUA, and a second group was formed by the wild populations SAH-S1, SAH-S2 and almost all the cultivated populations.

**Figure 4:**
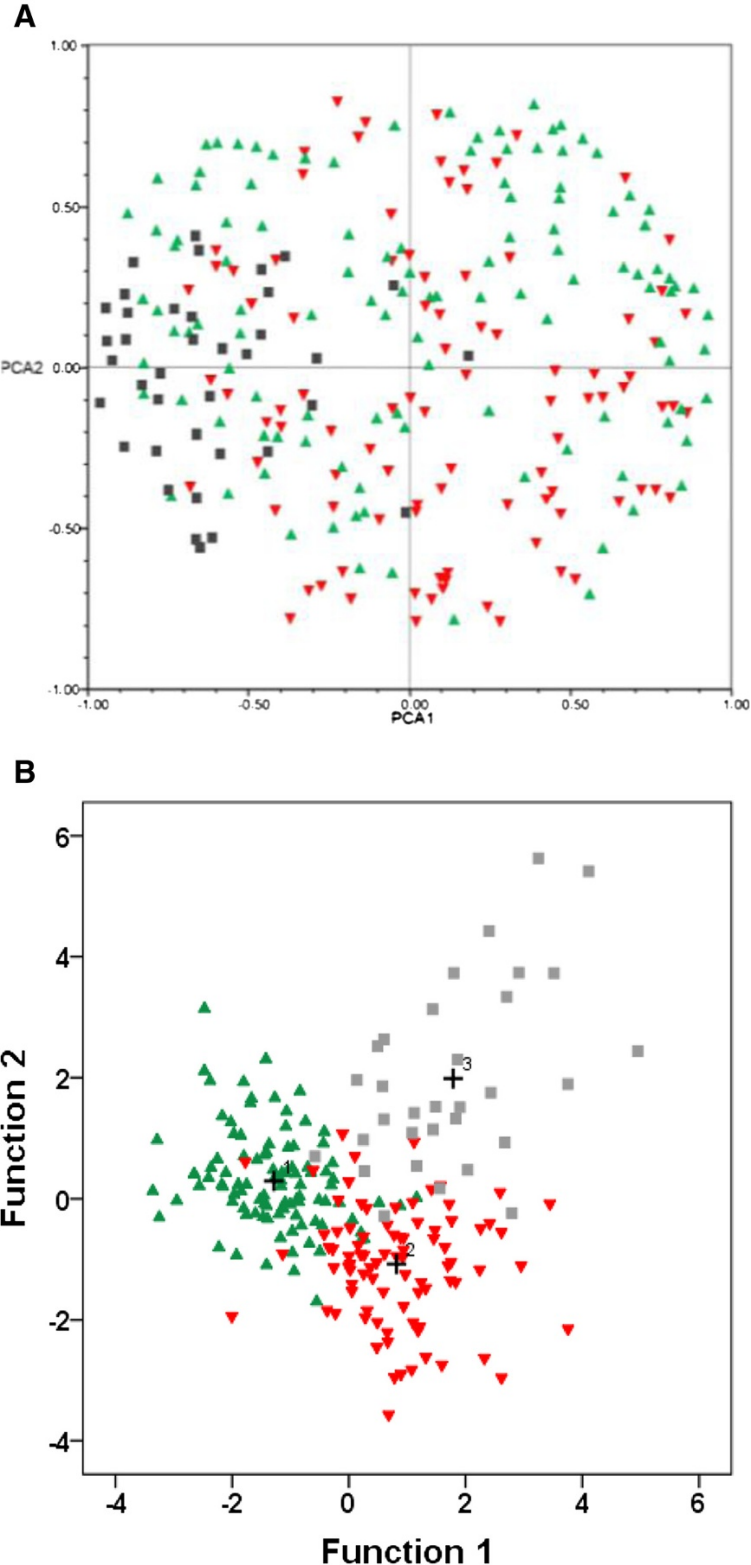
**Classification of individual plants of** ***Agave inaequidens*** **according to their type of management and** ***A. hookeri*** **(A) through principal component analysis, (B) through discriminant function analysis (DFA).** ▲ wild; ▼cultivated ■ A. *hookeri*, + centroid group.

The PCA analyzing the classification of individual agaves shows an overlap of individuals of the two species (Figure 4A). Wild plants of *A. inaequidens* are more abundant in the upper part of the plot and the cultivated ones in the lower part, whereas *A. hookeri* are grouped at the left of the plot, overlapping with some wild and cultivated individuals of *A. inaequidens*. The DFA resolves more clearly the morphological differences among wild and cultivated plants of *A. inaequidens* and those of *A. hookeri* (Figure 4B) and indicates that these differences were significant (Table 3) and associated to the plant, stem and leaf length (Table 1). Most individual were classified according to their category. Nearly 10% of the cultivated and wild plants are morphologically similar among themselves. None of the wild plants of *A. inaequidens* was classified as *A. hookeri*, while three of the cultivated plants were. Nearly 9% of plants of *A. hookeri* were classified within *A. inaequidens*, mainly the cultivated ones (Table 4).

**Table 3:** **Significance test of the Multivariate Analysis of Variance (MANOVA) among wild and cultivated population of** ***Agave inaequiens*** **and population of** ***A. hookeri***

Discriminant function	Auto value	% of variance			Canonic correlation
1	1.509	57.80			0.776
2	1.100	42.20			0.724
Contrast of function	Wilks’ Lambda	*Χ* ^*2*^	*F*	*df*	Significance
1 to 2	0.190	335.703	3.017	50	<0.001
2	0.476	149.851		24	<0.001

**Table 4:** **Classification of wild, cultivated of** ***Agave inaequidens*** **and** ***Agave hookeri*** **individuals according to the Discriminant Function Analysis (DFA)**

Actual group	Predicted group					
	***A. Inaequidens*** Wild		***A. inaequidens*** Cultivated		***A. hookeri***	
	N	%	N	%	N	%
*A. inaequidens* Wild	92	92	8	8	0	0
*A. inaequidens* Cultivated	8	9.3	75	87.2	3	3.5
*A. hookeri*	2	6.3	4	12.5	26	81.3

The patterns described support the fact that wild and cultivated populations of *A. inaequidens* are significantly different. One-way ANOVAs showed that these populations differ significantly in 13 of the 25 characters evaluated, and these are the most meaningful as identified by the eigenvectors of PCA. Populations of *A. hookeri* differed significantly in 23 of 25 characters with respect to the wild populations of *A. inaequidens*, while the cultivated populations of this latter species significantly differed from *A. hookeri* in only six characters.

## Discussion

The results described indicate that there is important morphological divergence between wild and cultivated populations of *A. inaequidens*, as well as among the populations of this species with those of *A. hookeri*. The main differences are those related to the plant size (the smaller being the wild plants of *A. inaequidens*, the largest being plants of *A. hookeri*) and spikiness (higher in the cultivated populations of *A. inaequidens*, the lowest in *A. hookeri*). These results are according to those hypothesized, as well as with those trends found in other species of agaves used for producing fermented and distilled beverages [22, 23, 34].

The patterns of morphological variation described may be result of management and artificial populations occur. However, it is relevant to observe that the differences are more clearly related to the management type than to the geographic location of the populations, which, although not concluding, suggests that management has a relevant influence in the morphological differentiation found in this study. Overlap of phenotypes in wild and cultivated populations are explained because artificial selection is basically increasing the frequency of ‘good’ phenotypes that occur at low frequency in wild populations.

The morphological differences found between *A. inaequidens* and *A. hookeri* support the proposal that these taxonomic entities may be different species. However, not all the individuals of these species remain as discrete different entities. The morphological is particularly high with plants from the cultivated populations of *A. inaequidens*. These patterns remain inconclusive. Similarities between *A. hookeri* with the cultivated populations could support our original hypothesis that *A. hookeri* derives from domestication of *A. inaequidens* and *A. hookeri* may be the extreme of a gradient of morphological trends associated to historical artificial selection. But also *A. inaequidens* and *A. hookeri* could be two different species and the morphological similarities shared by the cultivated plants of *A. inaequidens* and *A. hookeri* could be an expression of hybridization between the two taxa, since reproductive interactions between different species are rather common in the genus *Agave* [34–38].

The cultivated populations of *A. inaequidens* clearly had taller plants with longer stems and longer and wider leaves than the wild populations. Moreover, these features were even larger in *A. hookeri*. These are characters associated to domestication in agave and suggest that artificial selection is a cause of this morphological trend. Therefore, the plant size is apparently favoured at present while people select seeds from vigorous plants and then select the most vigorous plantlets in seed beds for their plantations. Plant size is clearly advantageous for people in terms of amount of sap moved within the plant tissue and therefore for producing ‘aguamiel’ and ‘pulque’. Also, it is advantageous for the ancient preparation of cooked stems consumed as food and for the current destiny of this matter for producing mescal. Larger individual plants may produce higher usable matter; however, it is still important to evaluate the concentration of sugar of both sap and plant tissue, because the quality (not only the quantity) of the useful product is in relation to this character. A large sized plant producing lower amount of sugar in its sap and or tissue is not necessarily a good resource. This fact has been documented at least with *Agave potatorum* by [39], in which it was found that smaller agaves are preferred over larger agaves since the smaller produce better quality mescal.

The spikiness shows a contradictory pattern since it is the highest in the cultivated *A. inaequidens* and the lowest in *A. hookeri*. In terms of management, lower spikiness is more favourable and this is clear in *A. hookeri*, the contradictory trend in *A. inaequidens* cannot be explained with the current information, since our interviews did not identify an explicit intention of reducing spines of the managed agaves. A feature associated to management that requires further analysis is concentration of saponins and other secondary compounds causing irritation of human skin. People that manage agaves continually suffer the effect of this chemical defence and they explicitly prefer agaves ‘mansos’ which are less irritant than ‘brutos’. Such preference would allow hypothesizing that agaves ‘mansos’ should have lower concentration of saponins in their tissue, but this is a supposition yet to be tested.

The success of propagation through vegetative parts is clear in *A. hookeri* but not in *A. inaequidens*. This feature is consistent with the supposition that *A. hookeri* is representative of the extreme of domestication of *A. inaequidens*. However, it is still necessary to know more about the weakness of vegetative propagules of *A. inaequidens* and the weakness of sexual propagules of *A. hookeri*. The absence of wild populations of *A. hookeri* and the weakness of its sexual propagules makes this species highly dependent from humans for its reproduction. It is therefore important to test the genetic relatedness between the two species analysed in this study, as well as to test the reproductive biology of *A. hookeri* and the viability of its seeds and plantlets. The failure of its sexual reproduction could be a testimony of its origin associated to human management, but this problem deserves further research.

People cultivating *A. inaequidens*, particularly those from Queréndaro form their plantations with agave plantlets coming from neighbouring zones (from both forests and plantations) but also from areas 100 to 200 km away. This fact may be contributing to maintain high morphological and genetic variation similarly as reported by [24] for *A. angustifolia*. In the case of *A. hookeri* people continually select vegetative propagules derived from the largest plants, and this is the simple principle of artificial selection maintained in this species to conform new plantations.

### Implications for conservation

Numerous species and varieties of agaves are currently of high cultural, social and economic importance, as well as important components of the ecosystems where they form part; some of them are key species of the biotic communities [35–38]. Although production of distilled beverages of agave is several centuries old, from the 19th Century the production of mescal and particularly that called tequila, has increasing demand, becoming along with fibres, the most important products of this species group worldwide [40]. Mescal production involves nearly 50 species of *Agave*, most of them extracted from the wild [38, 41], which commonly have considerable risk when demand of mescal increases in the market [41] and natural populations may be drastically affected and even getting locally extinct. Because the extraction of agaves for producing mescal occurs just before sexual reproduction, those species depending on this type of Reproduction for their population recovery are severely affected. This is the case of *A. inaequidens*.

In this study, the ethnobotanical information allowed documenting that plantations of *A. inaequidens* are commonly conformed to plantlets of differential provenance, which suggests that these plantations are reservoirs of diverse gene pools that may constitute valuable germplasm banks. Another important fact is that the size of the populations utilized is progressively decreasing. Local people indicated that they have to go farer away for collecting agaves for mescal production. Cultivation is emerging as an alternative for facing such progressive decreasing of natural populations but technologies for cultivation are still in construction. The local experience of agave management is undoubtedly an important source of techniques for rapidly developing the required techniques, but researchers have also an important role to accomplish, contributing to document and systematize the regional experience and carrying out experiments that allow answering questions associated to the processes of management.

In November 2012, a total of 29 municipalities of the state of Michoacán, Mexico received the Denomination of Origin (DO) of the mescal production from six species, among them *A. inaequidens*. It is indispensable the design and implementation of management strategies, particularly because the inclusion of the region in the Denomination of Origin may determine a drastic increase in the demand and production, endangering the survival of the already progressively scarcer natural populations of *A. inaequidens*.

Origins of *A. hookeri* remains being a mystery. Our current data support the proposal by [28] that it may have derived from domestication of *A. inaequidens*. Further morphological studies including reproductive parts and molecular genetics may provide light in this respect. However, in terms of conservation biology, it is urgent a strategy for identifying the remaining areas with the presence of this species. Our current sampling confirmed that populations are scarce and that are particularly vulnerable since the demand of pulque from this species is apparently decreasing. The loss of the regional culture of pulque consumption would determine the loss of cultivation of this apparently rare taxon.

## Authors’ information

CJF postgraduate student at the Centro de Investigaciones en Ecosistemas (CIEco), UNAM. AC, and AGR full time researchers at CIEco, UNAM. PCGM researcher at the Centro de Investigaciones Científicas de Ycatán, Mexico. JMN researcher at the Instituto Venezolano de Investigaciones Científicas.
